# Executing plans to enhance diversity across cancer centers in the United States: opportunities and challenges

**DOI:** 10.1093/jnci/djae100

**Published:** 2024-05-06

**Authors:** Christopher I Li, Sherise Chantell Rogers, Carol J Bult, Carmen E Guerra, Angela Talton, Lovoria B Williams, Wendy Law

**Affiliations:** Fred Hutchinson Cancer Center, Seattle, WA, USA; Fred Hutch/University of Washington/Seattle Children’s Cancer Consortium, Seattle, WA, USA; University of Florida, Gainesville, FL, USA; The Jackson Laboratory, Bar Harbor, ME, USA; Abramson Cancer Center, University of Pennsylvania, Philadelphia, PA, USA; City of Hope, Duarte, CA, USA; University of Kentucky College of Nursing & Markey Comprehensive Cancer Center, Lexington, KY, USA; Fred Hutchinson Cancer Center, Seattle, WA, USA; Fred Hutch/University of Washington/Seattle Children’s Cancer Consortium, Seattle, WA, USA

## Abstract

**Background:**

Lack of diversity in the cancer research workforce persists, which the new requirement for all National Cancer Institute (NCI)–designated cancer centers to have a Plan to Enhance Diversity (PED) seeks to address. However, it is not well understood how different cancer centers are approaching the development and execution of these plans. Our objective was to assess how cancer centers are establishing and pursuing their PED.

**Methods:**

We conducted a cross-sectional survey of members of the Cancer Center Diversity, Equity and Inclusion Network, which includes all NCI-designated cancer centers and several emerging centers. A total of 62 cancer centers (75% of those invited), including 58 NCI-designated cancer centers (81% of those with this designation), participated and completed a questionnaire that assessed PED leadership, major challenges, implementation strategies, and approach to evaluate PED progress.

**Results:**

The most common PED challenge identified is recruiting diverse faculty (68% of centers), and the most common strategy currently used to address this is reviewing and revising faculty recruitment practices (67%). The most common approach centers are using to measure PED progress is shifts in demographics (68%), and data on the demographics of faculty, leadership, and trainees are available at 79%, 81%, and 75% of centers, respectively.

**Conclusions:**

Almost all centers have established a PED leadership structure, however, there is considerable variation in the approaches used to realize PED goals and in the resources provided to support PED work. Realizing opportunities to share and implement common best practices and exemplar programs has the potential to elevate the impact of PED efforts nationally.

As detailed in the 2023 report from the National Academies entitled, *Advancing Antiracism, Diversity, Equity, and Inclusion in STEMM Organizations: Beyond Broadening Participation*, there remains a persistent lack of diversity among academic scientists and physicians conducting biomedical research. This is evident in the field of cancer research where this underrepresentation is also present at the leadership level. According to the findings of Lerman and colleagues (1), the majority of the directors of the nation’s National Cancer Institute (NCI)–designated cancer centers are non-Hispanic White (79%) and male (84%). These data stand in stark contrast to an increasingly diverse United States where cancer is the leading cause of death among Americans aged younger than 85 years (2) and marked racial and ethnic disparities in cancer incidence and mortality continue to persist (3). The tremendous values gained from diverse workforces have been extensively documented as diversity has been shown to increase teamwork, creativity, problem solving, innovation, productivity, and loyalty (4). For example, diverse scientific teams publish more and are cited more frequently than less diverse teams, and in medicine, diversity in health professional schools not only improves community health but also increases the understanding of different perspectives and decreases bias (5-7).

Integrating data from several sources, Table 1 summarizes the demographics of the cancer research workforce in the United States. Particularly stark is the substantial underrepresentation of American Indian and Alaska Native, Black, and Hispanic people from medical students to cancer center directors and the low proportion of women who are cancer center directors.

**Table 1.** Demographics of the cancer research workforce in the United States

Demographics	US population,^a^ %	Medical school matriculants, ^b^ %	Oncology fellows, ^c^^,^^d^%	Active physicians,^e–f^ %	Cancer center leadership,^e^ %	Cancer center directors,^e^^,^^g^ %
Women	50.8	55.4	48.0	35.9	36.3	14.0
Race and ethnicity						
American Indian or Alaskan Native	1.3	0.2		0.3		0.0
Asian	5.9	22.7		17.1	11.0	10.2^h^
Black	13.4	9.4	4.0	5.0	3.5	5.1
Hispanic	18.5	7.0	5.0	5.8	3.8	6.8
Native Hawaiian or Pacific Islander	0.2	0.1		1.0		not reported
White	76.3	42.3		56.2	82.2	76.3

a Data adapted from US Census 2019 US Census Bureau QuickFacts: United States. https://www.census.gov/quickfacts/US and accessed on November 28, 2023.

b Data adapted from Association of American Medical Colleges Diversity in Medicine: Facts and Figures 2019, https://www.aamc.org/data-reports/workforce/report/diversity-medicine-facts-and-figures-2019, accessed on November 28, 2023.

c Data adapted from Winkfield KM, Flowers CR, Patel JD, et al. (2017). American Society of Clinical Oncology strategic plan for increasing racial and ethnic diversity in the oncology workforce. *J Clin Oncol*. 2017;35(22):2576-2579. https://doi.org/10.1200/JCO.2017.73.1372 and https://www.asco.org/sites/new-www.asco.org/files/content-files/practice-and-guidelines/documents/2020-workforce-information-system.pdf.

d Data adapted from 2021 Snapshot: State of the Oncology Workforce in America. *JCO Oncol Pract*. 2021;17(5):249. https://ascopubs.org.

e Data adapted from Morgan A, Shah K, Tran K, et al. Racial, ethnic, and gender representation in leadership positions at National Cancer Institute–designated cancer centers. *JAMA Netw Open*. 2021; 4(6):e2112807. doi: 10.1001/jamanetworkopen.2021.12807.

f Data adapted from Association of American Medical Colleges Diversity in Medicine: Facts and Figures 2019, Figure 18. Percentage of all active physicians by race/ethnicity, 2018, https://www.aamc.org/data-reports/workforce/data/figure-18-percentage-all-active-physicians-race/ethnicity-2018, accessed on November 28, 2023.

g Data adapted from “Caryn Lerman’s Association of American Cancer Institutes (AACI) presidential initiative: Close the diversity gap in the cancer centers’ workforce”. Cancer Letter; 47(41), November 5, 2021.

h Includes Asian and Pacific Islanders.

The NCI has long demonstrated its commitment to diversity through supporting multiple programs to enhance workforce diversity at all levels, particularly through its Center to Reduce Cancer Health Disparities. To accelerate progress in this area, in 2021 NCI began requiring all NCI-designated cancer centers to design and execute a Plan to Enhance Diversity (PED) as a new core component of the Cancer Center Support Grant (CCSG) application. Per PAR-21-321, each center’s PED must address the following 5 elements.

1. Enhance participation of women, minorities, and individuals from groups nationally underrepresented in the research workforce, center leadership, and advisory boards while using special opportunities, if available, within the center’s catchment area to enhance the diversity of the research workforce and center leadership of CCSG-supported components.
2. Support career-enhancing research opportunities for junior, early, and mid-career researchers, including those from diverse backgrounds, to prepare them for center leadership.
3. Establish infrastructure and utilize institutional resources to expand the pipeline of members of diverse backgrounds through training or mentoring opportunities to encourage participation of students, postdoctoral researchers, and co-investigators from diverse backgrounds, including those groups shown to be nationally underrepresented in the research workforce.
4. Leverage institutional commitment and infrastructure to enhance diversity of the center’s membership and leadership.
5. Establish criteria for monitoring and evaluating the progress of diversity.

In response to addressing this new component, cancer centers began to name associate directors for diversity, equity, and inclusion (or equivalent) to develop a PED and invite them to join their respective cancer center leadership teams. To initiate national conversations on how to approach PED development and execution, the Cancer Center Diversity, Equity, and Inclusion (DEI) Network was formed by the Fred Hutch/University of Washington, Seattle Children’s Cancer Consortium, a NCI-designated comprehensive cancer center, in October 2021 to provide a forum for PED leaders to share best practices, develop common metrics for tracking and reporting PED progress, identify common issues and challenges, and share exemplar PED relevant programs and activities. This network has grown rapidly and now includes representatives from 100% of the nation’s NCI-designated cancer centers as well as several emerging centers.

Given the newness of the PED component, and to help guide each center’s work, the Cancer Center DEI Network sought to systematically capture information on how PED is being led and executed across the nation. The network conducted a comprehensive survey to record information on PED leadership, resources, existing programs, existing data, opportunities, challenges, and how success is defined and evaluated. This manuscript describes the results of this PED survey and provides recommendations and guidance toward increasing the effectiveness and sustainability of PED initiatives. The results of this survey form a baseline against which future surveys can be compared to assess progress.

## Description of the Plan to Enhance Diversity questionnaire

The PED questionnaire was developed by the authors of this manuscript to assess 4 primary questions:

1. How is PED being led across cancer centers and what are the characteristics of PED leaders?
2. What are the perceived major challenges in PED development and implementation?
3. What strategies are cancer centers using in their PEDs?
4. How are cancer centers evaluating the progress and success of their PED efforts?

The 87-item electronic questionnaire was created using SurveyMonkey. It employed a series of primarily multiple choice questions with opportunities for open-ended and other text responses. This instrument was pilot tested by PED leaders not involved in the design of the survey and refined based on their feedback. Invitations to participate were sent to representatives of the 84 centers that comprised the membership of the Cancer Center DEI Network as of February 2023. Responses by cancer centers were tracked, and multiple reminders were sent to the representatives of centers who had not completed the survey. The survey was open from February to June 2023, and responses were received from 62 centers (75% response rate across all centers) including 58 of the 72 NCI-designated centers (81% response rate among NCI-designated centers).

## Characteristics of the responding cancer centers

The 62 participating cancer centers are in all regions of the United States (Table 2) and are geographically representative of the nation’s NCI cancer center program. The majority of individuals who completed this survey were the associate directors for PED at their cancer center (69%), and for the remaining centers, it was completed by a PED staff member, associate director for administration, or other cancer center staff member. Of the responding centers, 58 are NCI-designated cancer centers of which there are 3 designations: basic cancer center (n = 6), cancer center (n = 10), and comprehensive cancer center (n = 42). One way of describing a cancer center’s size is by the number of faculty who are members of the cancer center. Of the responding cancer centers, 62% had fewer than 200 members, and 22% had at least 300. Another assessment of the scope of a cancer center’s work is the population size of its geographic catchment area—the population it seeks to serve through its research, clinical, and community-based efforts. Excluding the 6 basic science centers that are not required by NCI to define a catchment area and the 4 not NCI-designated centers, 58% of responding cancer centers serve a population of more than 5 million Americans with 23% serving more than 10 million and only 6% serving less than 2 million. Reflective of demographic variations across the nation, there were considerable differences in the catchment area proportion of individuals from underrepresented racial and ethnic minority populations per the National Institutes of Health (NIH) definition (which defines American Indian and Alaska Native, Black and African American, Hispanic, and Native Hawaiian and Pacific Islander people as underrepresented in the scientific workforce) across the regions participating cancer centers serve. Of the centers, 29% were in areas where less than 20% of the population is underrepresented racial and ethnic minority populations, and 23% were in areas where more than 50% of the population is underrepresented racial and ethnic minority populations.

**Table 2.** Characteristics of participating cancer centers

Characteristics	No. (%) (n = 62)
Geographic region	
Northeast	18 (29)
Southeast	12 (19)
Midwest	12 (19)
Southwest	6 (10)
West, Mountain West	14 (23)
Type of cancer center	
National Cancer Institute designated	58 (94)
Comprehensive	42 (68)
Cancer center	10 (16)
Basic center	6 (10)
Not National Cancer Institute designated	4 (6)
Number of cancer center members and faculty	
<100	7 (13)
100-199	31 (49)
200-299	11 (17)
300-499	10 (16)
≥500	4 (6)
Size of the catchment area population^a^	
<500 000	0 (0)
500 000-1 000 000	0 (0)
1 000 000-1 999 999	3 (6)
2 000 000-4 999 999	19 (37)
5 000 000-9 999 999	18 (35)
≥10 000 000	12 (23)
Proportion of the catchment area population that are underrepresented racial and ethnic minorities^a^	
<9%	6 (10)
10-19%	12 (19)
20-29%	11 (18)
30-39%	13 (21)
40-49%	6 (10)
≥50%	14 (23)

a Excludes basic centers and centers that are not designated by the National Cancer Institute (NCI).

## PED leadership and support

Of the cancer centers, 88% have an appointed PED leader, and at 91% of cancer centers, the PED leader participates on the center’s senior leadership team (Table 3). Of the centers that have named PED leaders, 77% of these leaders are women, 48% are Black, 27% are Hispanic, 21% are non-Hispanic White, and 17% are Asian (Table 3). Thus, PED leaders are considerably more diverse than cancer center leaders and cancer center directors who are 36% and 14% women, respectively, and 82% and 76% non-Hispanic White, respectively (1). We also captured additional dimensions of diversity and note that 29% of PED leaders are immigrants, 35% are the first in their family to go to college (first generation), 15% served in the military, 7% identify as lesbian, gay, bisexual, transgender, queer, intersex, and/or asexual (LGBTQIA+), and 7% have a disability. These characteristics did not vary appreciably by cancer center type or size.

**Table 3.** PED leadership and support^a^

Characteristic	All centers, % (n = 62)	All NCI-designated centers, % (n = 58)	NCI-designated Comprehensive Cancer Centers, % (n = 42)	Centers with <200 members, % (n = 36)	Centers with ≥200 members, % (n = 26)
PED leadership					
Have an appointed PED leader	88	94	93	85	92
PED leader serves on center’s executive committee or equivalent	91	90	90	97	82
PED leader demographics					
Female	77	75	76	81	70
Race and ethnicity					
Asian	17	18	18	18	15
Black	48	47	50	54	40
Hispanic	27	27	24	25	30
Non-Hispanic White	21	13	15	7	20
Any National Institutes of Health underrepresented category	80	78	79	79	80
First generation	35	35	34	37	32
Immigrant	29	27	26	25	35
LGBTQIA+	7	10	10	8	11
Disability	7	7	9	8	5
Served in the US military	15	14	16	19	10
Support for PED					
Full-time equivalent salary support for PED leader					
≤0.05	9	7	18	12	4
0.10	33	22	23	38	25
0.15	10	9	13	12	8
0.20	22	19	25	21	25
0.25-0.40	10	11	15	6	17
≥0.50	14	15	18	12	17
Level of full-time equivalent for PED staff					
0.0	17	19	10	18	17
0.5-0.85	14	15	18	9	21
1.0	33	30	30	44	17
1.1-1.5	14	13	15	11	17
2.0-2.5	9	9	10	3	17
≥3.0	14	15	18	14	13
PED advisory boards					
Have an internal advisory board	72	77	73	72	52
Have an internal and an external advisory board	19	20	17	24	12
Source of PED funds					
Institutional funds	86	87	88	88	83
NCI cancer center support grant	40	43	43	45	33
Philanthropy	33	36	33	36	29

a LGBTQIA+ = lesbian, gay, bisexual, transgender, queer, intersex, and/or asexual; NCI = National Cancer Institute; PED = Plan to Enhance Diversity.

There is considerable variation in the level of effort support that PED leaders are provided (range = 2%-80%). At 42% of centers, the PED leader has no more than 10% full-time equivalent in their salary support; at 24% of centers, the PED leader has at least 25% full-time equivalent. Similarly, there is a wide range of staffing levels to support PED work ranging from 0 staff to 5.0 full-time equivalent. Support staff full-time equivalent varied by size of the cancer center. At cancer centers with less than 200 members, 18% of PED leaders had at least 25% full-time equivalent of staff support compared with 34% of PED leaders at centers with at least 200 members. Of the centers, 17% have no PED support staff, and 14% have at least 3.0 full-time equivalent of PED support staff. Of the centers 70% have at least 1 full-time equivalent, and this varied somewhat by cancer center size. Whereas 73% of centers with less than 200 members had at least 1.0 full-time equivalent of PED staff vs only 62% of centers with more than 200 members, 30% of centers with at least 200 members had more than 2.0 full-time equivalent of PED staff compared with 17% of centers with less than 200 members. To help guide their work, 72% of centers have established an internal PED advisory committee, and of these, 19% also have a PED-specific external advisory committee.

## PED implementation: major challenges and key strategies

Although efforts focused on advancing a climate and culture of inclusive excellence and supporting diversity, equity, and inclusion at cancer centers and their associated and partnering academic institutions are not new, the structure and required elements of PED are new, and few centers had existing leaders or infrastructure centered on this work. As such, cancer centers across the country are at different stages of initiation, implementation, and sustainability of their PED-related efforts. Our survey sought to identify and measure the PED challenges that cancer centers currently face by asking centers to report their top 3 challenges. Only 1 challenge was reported by more than half of the centers: recruiting diverse faculty (68%). The other PED-related challenges reported by more than 15% of centers include obtaining DEI-related data and metrics (49%), shifting institutional climate and culture (31%), increasing leadership diversity (27%), lacking sufficient resources (25%), retaining diverse faculty (22%), recruiting diverse trainees (20%), dismantling structural racism and bias (17%), and supporting diverse faculty (15%). Below we describe cancer centers’ current and planned strategies to address a number of these challenges.

### Current and planned strategies to enhance the diversity of cancer center members and faculty

To enhance faculty diversity at cancer centers, there are several current strategies being used nationwide (Table 4), many of which are evidence-based best practices. Faculty development and mentoring of those underrepresented in health care and biomedical research are important approaches (8). Strategies include supporting career development through initiatives such as advanced skills in teaching, writing for publication, and designated pilot funding for research. Other demonstratable efforts include larger systemic policy changes. For example, 67% of cancer centers are reviewing or revising their hiring and recruitment practices. One specific, higher resource strategy being used by 28% of centers is faculty cluster hiring, which involves recruiting a diverse cohort of faculty across departments simultaneously instead of the more traditional approach of individual faculty hiring within a department (9,10).

**Table 4.** Current and planned strategies to enhance the diversity of faculty, leadership, and trainees

**Strategies to enhance faculty diversity**	
Current strategies	Planned future strategies
Tier 1 (≥50%) 67% review/revise faculty recruitment practices Tier 2 (20%-49%) 38% review and/or revise academic policies 33% career development for URSW^a^ faculty 33% enhanced mentoring for URSW faculty 33% designated pilot funds for URSW faculty 31% identity-based faculty affinity groups 28% faculty cluster hiring Tier 3 (10%-19%) 14% grant writing workshop for URSW faculty 14% bridge funding for URSW faculty 10% sponsorship program for URSW faculty 10% endowed chairs for URSW faculty	Tier 1 (≥50%) 77% career development for URSW faculty 68% enhanced mentoring for URSW faculty 58% designated pilot funds for URSW faculty 53% review/revise faculty recruitment practices Tier 2 (20%-49%) 42% review and/or revise academic policies 35% sponsorship program for URSW faculty 33% identity-based faculty affinity groups 33% faculty cluster hiring 32% grant writing workshop for URSW faculty Tier 3 (10%-19%) 23% bridge funding for URSW faculty 19% endowed chairs for URSW faculty
**Strategies to enhance leadership diversity**	
Current strategies	Planned future strategies
Tier 2 (20%-49%) 46% created new assistant and/or deputy leader roles 33% leadership-specific mentoring for URSW faculty 27% institutional leadership succession planning 25% leadership training program Tier 3 (<20%) 2% term limits for leaders	Tier 1 (≥50%) 74% leadership-specific mentoring for URSW faculty 68% leadership training program Tier 2 (20%-49%) 44% institutional leadership succession planning 37% created new assistant and/or deputy leader roles Tier 3 (<20%) 14% term limits for leaders
**Current and planned future pathway programs to enhance trainee diversity at different levels**	
Current URSW-specific pathway programs	Planned future URSW-specific pathway programs
Tier 1 (≥50%) 93% high school students 91% undergraduates 71% graduate students 62% postdoctoral fellows 58% postbaccalaureate scholars Tier 2 (20%-49%) 40% middle and/or junior high school students Tier 3 (<20%) 16% elementary school students	Tier 1 (≥50%) 79% postdoctoral fellows 70% graduate students 60% undergraduates 53% postbaccalaureate scholars Tier 2 (20%-49%) 47% high school students 40% middle and/or junior high school students Tier 3 (<20%) 15% elementary school students

a URSW = underrepresented in the scientific workforce.

Efforts to support the career advancement and retention of underrepresented faculty are also critical. At present, approximately one-third of centers have programs specifically to support the career development and enhance the mentoring of underrepresented in the scientific workforce faculty [based on the current NIH definition (11), which includes people from underrepresented racial and ethnic groups, individuals with disabilities, and those from socioeconomically disadvantaged backgrounds], including designating pilot funds specifically for these individuals. Encouragingly, most centers have plans to initiate efforts to enhance career development (77%), mentoring (68%), and accessibility of pilot funds (58%) for underrepresented in the scientific workforce faculty. Additionally, 35% of centers plan to develop a sponsorship program for underrepresented in the scientific workforce faculty, 23% plan to enhance their bridge funding, and 19% plan to create endowed chairs to aid in the retention of underrepresented in the scientific workforce faculty. As an effort to increase a sense of community and belonging, 31% of centers have existing identity-based faculty affinity groups, and 33% plan to form these in the future.

#### Current and planned strategies to enhance the diversity of cancer center leadership

At present, there is no single approach being used across cancer centers to enhance leadership diversity, though with respect to future plans, 74% plan to develop leadership-specific mentoring for underrepresented in the scientific workforce faculty, and 68% plan to launch leadership training programs. Implementing term limits for leaders remains a strategy that few centers currently use (2%) or plan to use (14%).

#### Current and planned strategies to enhance the diversity of trainees

Driven by the central importance of education and training, and the requirement of all NCI-designated cancer centers to have a Cancer Research Training and Education Coordination (CRTEC) program, almost all centers have existing pathway programs focused on high school students (93%) and undergraduates (91%) with the majority also having programs for graduate students (71%), postdoctoral fellows (62%), and postbaccalaureate scholars (58%). Of note, the majority also plans to develop new programs to enhance the diversity of postdoctoral fellows (79%), graduate students (70%), undergraduates (60%), and postbaccalaureate scholars (53%).

### Engagement of PED with other CCSG components

To be successful, it is critical for PED leaders to be integrated with several of the cancer center’s key components. Of central importance is engagement with 2 other core CCSG components: the offices of Community Outreach and Engagement (COE) and Cancer Research Training and Education Coordination (CRTEC), which are 2 required CCSG elements (note that COE is not a component of NCI-designated basic centers). However, among the NCI-designated centers (excluding basic centers), only 55% have PED programs that have regular joint meetings with their center’s COE and CRTEC leaders. Specific collaborative and synergistic activities across PED, COE, and CRTEC are now expected by CCSG site visit review teams and may include activities such as partnership between PED and CRTEC on enhancing the diversity of trainee pathway programs and identification of needs for faculty recruitment to address disparities experienced by catchment area priority populations between PED and COE. Additionally, only 33% of PED leaders have regular meetings with research program leaders and/or PED-designated liaisons or representatives from research programs.

### Strategies to impact institutional climate and culture

The most common strategy cancer centers are using to impact institutional climate and culture is through providing optional educational opportunities for employees to enhance their understanding of diversity, equity, and inclusion (73%). Additionally, 64% of centers have employee resource groups, and 42% have identity-based affinity groups. With respect to required implicit bias-related training, 55% require this for all faculty search committees, 29% for cancer center leaders, 27% for all cancer center employees, and 25% for cancer center faculty. In terms of collecting information from faculty on their satisfaction and perceptions of institutional climate and culture, only 60% of cancer centers survey their faculty in this way with 32% conducting annual surveys, 26% surveying every 2-5 years, and 2% every 5 years or more. Of those that perform this type of surveying, 57% use a custom instrument, 14% use the Association of American Medical Colleges instrument, and 8% use the Collaborative on Academic Careers in Higher Education survey from the Harvard School of Education.

### Defining and measuring PED progress

Approaches that cancer centers are using to measure PED progress include shifts in demographics (68%), measures of institutional climate and culture (65%), shifts in pathway program demographics (53%), policy adherence (47%), and progress on strategic goals (47%). However, 26% of centers have yet to establish how they will define the success of their PED efforts. Although 2 of the most common ways that cancer centers will measure progress relate to assessing shifts in their demographics, there is considerable variability in the availability of demographic data across cancer centers. Data on cancer center members (faculty) and leadership are available at 79% and 81% of centers, respectively, where data are mostly obtained from their human resources department (70%) (Table 5); 75% also have data on trainees and 70% have data on their staff and workforce. Of those with available data, all have data on sex, and 93% have data on race and ethnicity. However, few centers have data on other dimensions of diversity such as disability, sexual orientation, gender identity, and the NIH definitions of socioeconomic disadvantage. Additionally, less than half of centers have data on other PED-relevant demographics such as applicants to faculty searches (42%), faculty promotion and retention rates (35%), and the membership of their external advisory board (38%).

**Table 5.** Availability of demographic data at cancer centers

	Percent for whom any demographic data are available, %	Source		Type of data available			
Category		Human resources, %	Individual survey, %	Race and ethnicity, %	Disability, %	Sexual orientation and gender identity, %	National Institutes of Health–defined categories of socioeconomic disadvantage, %
Faculty, members	79	70	20	93	39	15	13
Applicants to faculty searches	42	75	25	83	43	4	4
Faculty promotion and retention rates	35	75	15	95	45	0	5
Leadership	81	50	37	96	39	20	17
External advisory board	38	0	62	95	14	14	5
Trainees	75	36	40	97	40	7	24
Staff, workforce	70	82	13	92	44	not collected	8

### Summary and recommendations

Based on this nationally representative sample of NCI-designated cancer centers, there is heterogeneity in the most common challenges that cancer centers are facing in the implementation of their PED. A most common challenge reported by 68% of cancer centers is increasing faculty diversity, which centers have numerous current and planned strategies to address including revising faculty recruitment practices and enhancing the support of underrepresented in the scientific workforce faculty through improved mentoring, career development programming, and designation of pilot funds. However, more resource intensive interventions such as use of faculty cluster hiring and allocating bridge funding or endowed chairs for underrepresented in the scientific workforce faculty are planned to be used by one-third or less of cancer centers.

The second most reported challenge is obtaining, integrating, and reporting on key PED-related metrics. Encouragingly, more than 75% of centers now have available demographic data— primarily limited to sex, race, and ethnicity—on their faculty, leadership, and trainees, but few have data on other dimensions of diversity or metrics related to the demographics of their faculty applicant pools or faculty promotion and retention rates. Also, only 60% of centers have an existing strategy in place to conduct assessments of faculty satisfaction to evaluate their progress in fostering an institutional climate of inclusive excellence. The cancer center community is also hampered by the lack of a uniform instrument or approach for evaluating climate and culture making it difficult to compare current status and progress across centers.

The third most commonly reported challenge related to enhancing the diversity of cancer center leadership and trainees are, with respect to leadership, efforts are mostly nascent across cancer centers with no single strategy used by more than half of centers, but 74% of centers plan to develop leadership-focused mentorship for underrepresented in the scientific workforce faculty, and 68% plan to initiate leadership training programs.

Additionally, with respect to trainees, the vast majority of centers have existing programs at 1 or more levels to enhance the diversity of the next generation of researchers and health-care professionals. There is abundant evidence supporting the importance of the diversification of pathway programs. In particular, these efforts are critical to increasing the diversity of the physician, health professional, and medical scientist workforce, and with these enhancements, the alleviation of health disparities may be realized in marginalized communities (12-14). A recent study by Snyder et al. (15) showed that communities with higher Black primary care physicians were associated with decreased mortality rates for not only Black patients but also White patients regardless of whether they were treated by that physician. These data support the direct and indirect methods in which health equity can be realized by diversifying the face of health care (15). Capers et al. (16) explains that although postbaccalaureate programs intended to support the professional development of students underrepresented in medicine have demonstratable success for increased matriculation into medical school, we miss the larger population of minoritized groups earlier in the pipeline. Systemic inequalities and racism create barricades and ongoing impediments to entry and successful navigation to the journey. Students from underrepresented groups are less likely to have access to specialized gifted and talented programs, are more likely to have lower expectations from teachers, attend schools with less educational resources, and face increased disciplinary action for similar behaviors of their White counterparts (17). Therefore, a convincing argument is made that diversifying our health-care workforce requires earlier interventions, and the investments that the considerable majority of cancer centers are making in this area will hopefully have a substantial long-term impact. It is important to note that this survey was conducted prior to the US Supreme Court’s 2023 ruling that ended affirmative action in higher education (*Studentsfor Fair Admissions, Inc. [SFFA] v President and Fellows of Harvard College* and *SFFA v University of North Carolina*). This ruling will pose new challenges to the work of PED that are still being realized.

Striking differences were observed regarding the level of support cancer centers are investing in PED efforts as evidenced by the highly variable level of full-time equivalent support provided to PED leaders and the considerable range of staff full-time equivalent to support PED activities. Additionally, interactions between PED and other cancer center components are not formalized at most centers with only 46% having regular interactions between PED leaders and CRTEC and COE leaders and only 33% with research program leaders and representatives.

Based on our interpretation of the results of this survey and the existing landscape, and to optimally carry out the goals of the PAR, we recommend the following for all cancer centers.

### Leadership

- The PED leader must be a part of the cancer center’s executive leadership team (9% are not) and should ideally directly report to the center director.
- Cancer center leadership teams need to define what PED success looks like, be active and engaged partners in the success of PED goals, and hold joint accountability for their achievement.
- Cancer centers should establish an internal and external DEI advisory board to help guide and monitor the progress of their PED work.
- All cancer centers have external advisory boards to provide them with critical guidance on all aspects of their centers. These external advisory boards should include at least 1-2 members with specific expertise in diversity, equity, and inclusion work.
- Many PED leaders are new to the leadership of diversity, equity, and inclusion efforts, and many are also early career faculty members. Cancer centers must invest in the leadership development of their PED leaders to enhance their abilities to be successful.

### Resources

- To achieve the multiple requirements of a successful PED, PED leaders need sufficient time and effort allocated to support their work (41% have ≤10% full-time equivalent) and a team of staff to execute the PED (30% have <1.0 full-time equivalent). Resources are also needed for recruitment initiatives across trainees, faculty, and leadership; to enhance the diversity of training programs; to support retention; and to support career advancement and facilitate promotion.
- To be successful, PED teams need to have access to demographic data on the center’s faculty, leadership, and trainees, and efforts should be made to ascertain dimensions of diversity beyond sex, race, and ethnicity.

### Key PED components and activities

- Integration of PED efforts with other components of the cancer center is critical. At a minimum, PED leadership should hold joint meetings with COE and CRTEC leadership and should establish means of interfacing with each of the center’s research programs.
- Centers should continue to adopt evidence-based best practices to reduce bias and structural racism and enhance the diversity of applicant pools for faculty and leadership positions.
- Centers should use data to inform their progress and to guide activities aimed at enhancing their diversity and inclusion. Efforts should focus on the groups currently prioritized by the PAR: members and faculty, research staff, leadership, and trainees.
- Centers should consider regularly reviewing and revising academic policies and practices to reduce bias and sources of structural inequities.
- Centers should use structured and validated instruments to evaluate institutional climate and culture to identify gaps, inform institutional priorities, and assess progress over time.

### PED review

- Site visit teams organized by NCI to conduct CCSG reviews should include reviewers with specific expertise in diversity, equity, and inclusion and in the delivery of effective PEDs. PED reviews should align with the criteria stated in the CCSG PAR.

## Data Availability

The source of the data reported in this manuscript was a questionnaire that was voluntarily completed by leaders at different cancer centers. This effort was determined to not be research and thus was exempt from institutional review board review. As a part of questionnaire completion, respondents agreed to provide identifiable information under the condition that their institution’s data would not be shared in an identifiable manner. Outside parties interested in these data can contact Dr Li, and all reasonable efforts will be made to provide a de-identified dataset for the intended purposes proposed with the approval of the co-authors of this manuscript.
